# The Effects of a “Health at Every Size^®^”-Based Approach in Obese Women: A Pilot-Trial of the “Health and Wellness in Obesity” Study

**DOI:** 10.3389/fnut.2015.00034

**Published:** 2015-10-27

**Authors:** Mariana Dimitrov Ulian, Fabiana B. Benatti, Patricia Lopes de Campos-Ferraz, Odilon J. Roble, Ramiro Fernandez Unsain, Priscila de Morais Sato, Bruna Cristina Brito, Karina Akemi Murakawa, Bruno T. Modesto, Luiz Aburad, Rômulo Bertuzzi, Antonio H. Lancha, Bruno Gualano, Fernanda B. Scagliusi

**Affiliations:** ^1^Department of Nutrition, Faculty of Public Health, University of São Paulo, São Paulo, Brazil; ^2^School of Physical Education and Sport, University of São Paulo, São Paulo, Brazil; ^3^Faculty of Applied Sciences, State University of Campinas, Limeira, Brazil; ^4^Faculty of Physical Education, State University of Campinas, Campinas, Brazil; ^5^Faculty of Philosophy and Letters, National University of Buenos Aires, Buenos Aires, Argentina; ^6^Institute of Health and Society, Federal University of São Paulo, Santos, Brazil

**Keywords:** obesity, non-dieting intervention, Health at Every Size, mixed method, multidisciplinary intervention

## Abstract

This study explored the effects of Health at Every Size^®^-based intervention on obese women by qualitatively evaluating participants’ perception toward the program and quantitatively evaluating changes related to psychological, behavioral, and body composition assessments. A prospective 1-year quasi-experimental mixed-method trial was conducted. The mixed-method design was characterized by a spiral method, and quantitative and qualitative findings were combined during the interpretation phase. The qualitative data involved three focus groups; and quantitative data comprised physiological, psychological, and behavioral assessments. Initially, 30 participants were recruited; 14 concluded the intervention. From the focus groups, the following interpretative axes were constructed: the intervention as a period of discoveries; shifting parameters: psychological, physical, and behavioral changes; eating changes, and; redefining success. Body weight, body mass index, total body fat mass, and body fat percentage were significantly decreased after the intervention (−3.6, −3.2, −13.0, and −11.1%, respectively; *p* ≤ 0.05, within-time effect). Participants reported to be more physically active and perceiving better their bodies. Eating-wise, participants reported that the hunger and satiety cues and the consumption of more frequent meals facilitated their eating changes. Finally, participants reported that they could identify feelings with eating choices and refrain from the restrained behavior. These qualitative improvements were accompanied by modest but significant improvements in quantitative assessments. Clinicaltrials.gov registration: NCT02102061.

## Introduction

The prevalence of obesity has dramatically increased globally, affecting both developed and developing countries. It is estimated that in 2015, 2.3 billion adults worldwide will be overweight (body mass index, BMI ≥25 to 29.9 kg/m^2^) and nearly 700 million obese (BMI ≥30 kg/m^2^) (1). In Brazil, 12.4% of male and 16.9% of female adults are obese (2). Considering the well-known association of obesity with various health problems, the prevention and treatment of this condition have a major impact on public health actions and policies, which often have weight loss as the primary target and indicator of “treatment success” (3, 4).

In this context, restrictive diets are the cornerstone of the so-called “obesity treatment” (5). Studies have shown the potential of such interventions for obese. Illustratively, Barte et al. (6) did a systematic review to evaluate the effects of interventions with a dietary as well as a physical activity component aiming at weight loss. Twenty-two interventions that included healthy adults with a BMI between 25 and 40 kg/m^2^ were selected. Also, these interventions provided at least five contact sessions guided by a professional health care provider. Weight change data of 2,431 participants who completed the 1-year follow-up were available. At baseline, the mean initial BMI was 32.2 kg/m^2^. The mean effects of weight change was −4.3 kg [95% confidence interval (CI), −3.0 to −5.6 kg]; the mean effects of BMI change was −1.6 kg kg/m^2^ (95% CI, −1.1 to −2.0 kg/m^2^) and the mean effects of percentage weight change was −5.0% (95% CI, −3.6 to −6.5%). The authors concluded that these interventions are appropriate for participants with a BMI between 25 and 40 kg/m^2^. Similarly, Johnston et al. (7) determined weight-loss outcomes for popular diets based on diet class (macronutrient composition) and trade-marked diets. Weight loss was observed with any low-carbohydrate or low-fat diet and, because of this result, the authors supported the practice of any diet in order to lose weight. Nonetheless, although diets have been consistently shown to lead to short-term weight loss, maintenance of this loss is only achieved in approximately 20% of the cases (8). Additionally, some studies reporting the results of weight-loss programs have been published and raised important questions about their impacts on overall health, namely the triggering of binge eating, eating disorders, body dissatisfaction, and low self-esteem (1, 9). Finally, a great ethical concern regarding such programs has been brought to attention, due to the commonly observed diet-induced processes of culpability, stigmatization, and the reduction of self-freedom (1, 9–12).

As a result, a growing number of different approaches that shift the focus from weight management to health promotion in obesity have emerged (known as “non-prescriptive interventions” that, as well as having the characteristics mentioned, do not prescribe a diet). Among these, the *Health at Every Size*^®^ (HAES^®^) philosophy has gained ground (Health At Every Size and HAES^®^ are registered trademarks of the Association for Size Diversity and Health). This philosophy encourages health behaviors for people of all body sizes, regardless of body weight (BW) changes (13), based on the following principles: (1) to recognize that health and well-being are multi-dimensional; (2) to encourage the construction of a positive self-image; (3) to accept and respect the diversity of body shapes and sizes; (4) to promote eating in a manner that balances individual nutritional needs, as well as hunger, satiety, appetite, and pleasure; and (5) to promote enjoyable and sustainable physical activities (3, 10, 13–17).

Katzer et al. (18) compared non-prescriptive interventions in terms of their impact on behavioral, psychological, and medical aspects of 225 overweight and obese women (considered in this study as a BMI ≥28 kg/m^2^), aged 25–68 years. Three groups were formed with the following purposes: the first focused on behavioral techniques, the second on healthy eating and physical activity, and the third received the same instructions of the second group, but followed them without the accompaniment of the research team. No weight change was observed in any of the groups. However, 1 year after the intervention, participants in the first group significantly improved their behavior and psychological aspects when compared with other groups, and were the only group whose eating choices improved (18). Furthermore, Bacon and Aphramor (10) conducted a review from six randomized controlled trials on non-prescriptive interventions based on HAES^®^, which either had a physical exercise program associated or encouraged physical exercises and assessed adherence via questionnaires. The authors concluded that the non-prescriptive interventions were capable of improving blood pressure, lipid profile, physical activity levels, disturbed eating behaviors, self-esteem, and body image of the participants, despite no significant weight loss.

Although quantitative studies have shown promising results of these interventions, evidence regarding its effects on psychological and behavioral assessments and body composition is still very limited and needs further investigation. Moreover, it is worth noting that many of the interventions included in these studies only involved nutritional/psychological group-format lectures whereas the intervention proposed in the present study included philosophical discussions, individual nutritional sessions, and supervised promotion of physical activity. Thus, it is conceivable to state that our intervention advances the current scenario involved in the HAES^®^ philosophy by introducing more diversified and specific strategies, which may better fit the complexity of the “health” construct. Finally, little is known regarding the experiences of individuals engaged in such interventions as to how they affect the participants expectations, their body and health perceptions, and, more importantly, their previous expectations toward nutrition and physical activity. In this respect, studies combining qualitative and quantitative approaches can be of great value considering that neither one alone can lead to a broad understanding of a phenomenon.

This study consisted of a pilot-trial that aimed to qualitatively and quantitatively explore the effects of a non-prescriptive multi-disciplinary program based on the HAES^®^ philosophy on obese women. We aimed to evaluate the perception of the participants toward the program, in addition to potential changes in psychological and behavioral assessments and body composition. It was hypothesized that this intervention would induce a positive effect on the participants’ physical, emotional, and social well-being, despite modest, if any, improvements in body composition.

## Materials and Methods

### Study Design

**Figure 1:**
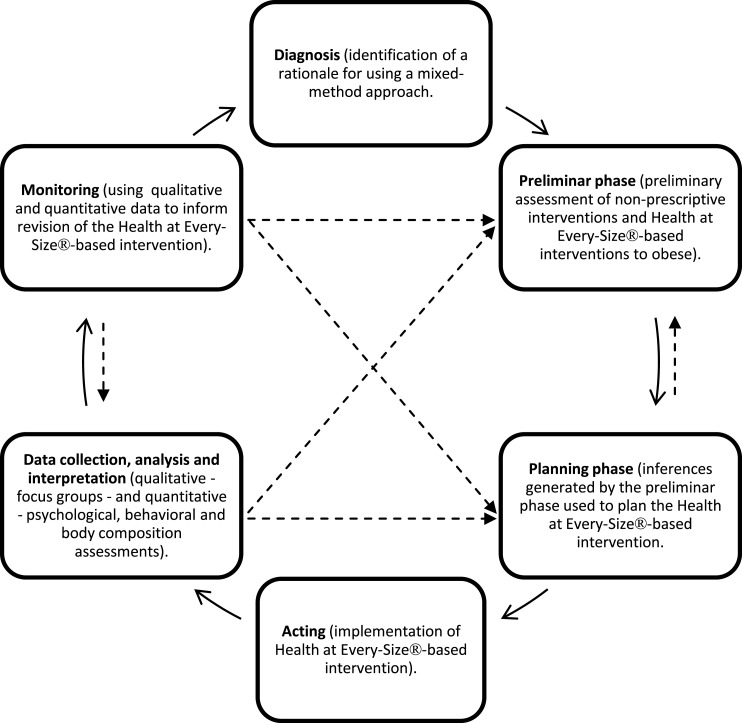
**Illustration of the conceptual framework**.

This pilot-trial was a prospective 1-year quasi-experimental mixed-method trial. The mixed-method design was characterized by a spiral method, as proposed by Borràs et al. (19). In this method, the study object is decomposed in different spheres, enabling the methodological planning, the formulation of hypothesis, and content for each of the spheres (e.g., diagnosis, planning, context, sample interaction, temporal dimensions). Throughout the intervention, this method encourages the use of partial results to deepen the findings, resulting in a spiral process that validates the spheres that compose the study’s object. The method enables the mutual and successive richness of both objective and subjective components of the phenomenon under study. Considering the present study objectives and research questions, emphasis was given on the qualitative data. The quantitative and qualitative findings were combined during the interpretative phase, in which quantitative findings were used to support the qualitative data. This conceptual framework is illustrated in Figure 1. The quantitative data indicate the effects of the intervention over health factors and the qualitative data support the understanding of the process that generated such effects and their meaning to the participants.

### Participants

**Figure 2:**
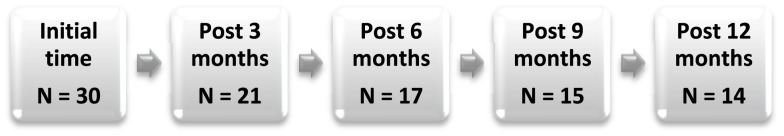
**Illustration of initial recruitment and the following attritions**.

The samples were the participants of the HAES^®^-based program called “Health and Wellness in Obesity Study,” which is a Community Program offered yearly at the School of Physical Education and Sport of the University of São Paulo, Brazil. They were recruited through the intervention being advertised via media ads. The inclusion criteria were women with 25–50 years old and BMI higher than 30 kg/m^2^. The exclusion criteria included illiteracy; type II diabetes; non-treated hypertension; current use of medications including appetite suppressants, thyroid hormone medication, orlistat, topiramate, or diuretics; evidence of thyroid, liver, lung, heart, or kidney disease; engagement in any form of regular exercise training programs; and engagement in any extra nutritional treatment (i.e., that was not part of the intervention). Initially, 30 participants were recruited, and 14 concluded the intervention. Figure 2 illustrates the initial number of participants and the following attritions.

The project was approved by the Ethics Committee of the concerned institution. The participants signed the informed consent, and all of the procedures were in accordance with the Helsinki Declaration revised in 2008. This is a pilot study from a trial registered at clinicaltrials.gov as NCT02102061, which aimed to advance our knowledge on the feasibility and efficacy of the proposed intervention.

### Characteristics of the Intervention

The intervention consisted of weekly physical activity sessions, five philosophical workshops, and bimonthly individual nutritional sessions. The physical activity sessions comprised aerobic exercises (i.e., walking, running, cycling, etc.), fun team sports and games (i.e., volleyball, handball, relay races, etc.), and strength exercises. Emphasis of the physical activities was given on the promotion of health, without focusing on the intensity, structure, and duration of the activity, or on the weight loss. The physical education professionals were prepared to identify fears, disappointments, shame, and other feelings that could arise from the participants during the physical activity sessions and to access them in an emphatic and collaborative way. Also, before commencing the physical activity sessions, and throughout the intervention, the participants could express their opinion on what kind of physical activity would be more enjoyable to them. Thus, the physical activity sessions were restructured when necessary. Additionally, when participants had a particular issue (e.g., physical injury), the physical educators provided them with individualized physical activities.

The philosophical workshops were conducted by one of the authors of this article (Odilon J. Roble), who has a bachelor degree in philosophy. He applied basic concepts of philosophy and encouraged participants to reflect. In the philosophical workshops, teasing-themes (i.e., discussions that triggered the talkings) related to desire and inner and outer expectations regarding weight, body, and appearance were introduced. These discussions aimed to provide the participants with tools that could help them gain new perspectives and change dysfunctional thoughts, and allowed them to construct their own reflections about body, control, decisions, and choices.

The nutritional intervention was based on nutritional counseling, which involved a supportive process that assists a person on how to better deal with their eating (if and when necessary) and enhance personal resources via strategies that foster the responsibility for self-care (20). The strategies used during the individual nutritional sessions comprised the use of the food record, the meal planning, and the setting of goals. All of these strategies were in accordance with the HAES^®^ principle that embraces promoting an eating that balances individual nutritional needs and that consider aspects such as hunger, satiety, and pleasure. More specifically, the meal planning aimed to promote a health eating by helping the participants regarding their eating structure and amounts eaten. It also involved providing a support plan for many different aspects, such as planning the grocery shopping, the preparation of the meals, the organizing of the pantry. The meal planning was proposed very gradually and was elaborated according to the most important aspect brought up by the participant during the nutritional session. The setting of goals involved proposing detailed strategies that would help the participants to achieve the changes that were meaningful to them. They were not exclusively related to eating changes: they involved any strategy from which the participants could benefit in their movement toward a more adequate eating (e.g., identifying people that could give support to them, brainstorming routine aspects that could be modified to facilitate a given change). With the strategies abovementioned, the participants were encouraged to increase their sensitivity to hunger and satiety cues, and to decrease their vulnerability to inner or outer triggers that lead to automatic behaviors related to food. Also, they were encouraged not to classify food dichotomously; to build a social support external to the intervention, and, finally, to find pleasurable activities and ways to be more active. Moreover, in the individual nutritional sessions, the nutritional therapists explored participants’ weight-loss expectation and whether it was an achievable goal. This was in order to address assumptions about weight stigma and discuss their consequences, giving the participants an opportunity to talk about their dieting history in an open and accepting environment (when and if it was the case). It was important to brainstorm why their dieting attempts did not work and to stress why the HAES^®^ philosophy do not encourage such strategies, and not to encourage weight-loss strategies. The nutritional therapists did not focus on weight as an outcome in any moment of the intervention. The participants were encouraged to develop a more positive relationship with food, to listen to their bodies, and to rely less on food to comfort them emotionally. They were also encouraged to explore other ways to self-nourish themselves than not with food; for example, by pursuing what they wanted to do regardless of weight, finding ways to look after themselves and investing in different roles they could play in life (e.g., building on other aspects of their lives rather than just being a spouse, a mother, a daughter, but also investing in other things that could stimulate and satisfy them, like going out and meeting friends, avoiding negative self-talk, pursuing a hobby, etc.). These individual sessions lasted for 45 min. Additional information regarding professionals’ training and contributions can be found in the Supplementary Material.

The intervention aimed to promote improvements on health and life, based on the idea that obese people can experience health and wellness regardless of whether they lose BW. The intervention coincided with the HAES^®^ philosophy by not aiming for the weight loss and not prescribing diets. Furthermore, the professionals involved were committed to its principles. Moreover, at the beginning and at the end of the intervention, anthropometric and body composition assessments were performed [comprising weight, waist and hip circumferences, fat mass (FM), and fat-free mass (FFM) assessments), and self-administered questionnaires and scales were administered. Finally, three focus groups, which were used for the qualitative assessment, were conducted throughout the intervention (the first was conducted in December/2012; post 4 months was conducted in April/2013; and the final was conducted in July/2013). Implementing separate focus groups was in accordance with the theoretical framework chosen – the action research, which consists of actions that aim to make specific changes to a given scenario from the perspective of those involved. The ongoing process of the focus groups allowed that the perspectives of the participants, expressed in the focus groups, guided the course of the intervention. In this context, focus groups were considered a strategy of data collection and a valued characteristic of the study.

### Qualitative Data Collection and Analysis

The qualitative data were undertaken with researchers not participating in the intervention to avoid an eventual bias (21). According to Liamputtong (21), the moderator should be a “neutral person” who can stimulate the participants and elicit responses. Thus, an experienced anthropologist conducted the three focus groups during the intervention and an observer wrote down expressions, gestures, and other non-verbal behaviors of the participants. Each focus group had its own guiding questionnaire. Considering that the activities and reflections of this intervention were gradually developed along its course, it was not appropriate to follow a standard guiding questionnaire for all the focus groups (21), because some questions would not be adequate or appropriate outside a given context. Thus, these guiding questionnaires were collectively constructed by all of the professionals. Moreover, they were not pre-tested, because they were specific to the characteristics of the obese people who participated in this particular study.

Although the focus group discussions covered issues relevant to all the areas involved in the intervention, the present article will examine only those related to the general experiences regarding the non-prescriptive program based on the HAES^®^ philosophy, as expressed in the first and third focus groups. The experiences regarding the nutritional intervention *per se* can be found elsewhere (22). Considering the scope of the present article, the focus groups explored how the intervention affected the participants’ lives and which changes they expected to maintain after the end of the intervention, specifically with respect to their physical, functional, emotional, and social well-being, and to having success in the treatment meant to them.

Liamputtong (21) argued that it is hard to maintain an active and interesting discussion in groups with less than four people, and that groups with more than eight people can be difficult to manage. Therefore, to ensure methodological rigor, seven to eight women were expected in each focus group. However, because we predicted possible absence of participants, 10 participants were invited and 7 participated in each focus group. The three focus groups were attended by 12 different women. Of these, two participated in three of the focus groups, five in two, and five in one focus group. Each session lasted from 80 to 100 min, were audio-recorded, and later transcribed verbatim. The observer’s notes were included in the transcriptions, though were not used in the final analysis. The observer’s notes were used for the professional team to observe the progress of the groups and the participants’ engagement in them. The data collected in the focus groups were shown to the participants at the end of the intervention.

The Collective Subject Discourse (CSD) technique was used for data analysis. It consists of procedures that allow for the organization of the discursive-nature qualitative data (23). The procedures involve, primarily, the selection of the most important excerpts of the speeches (called “key-expressions”). Subsequently, each “key-expression” receives a name that concisely describes its meaning (called “central idea,” CI). Finally, CIs that are similar or complementary are identified and their correspondent key expressions are gathered in a summing discourse, which is the CSD (23). This analysis was be performed by an experienced researcher and was independently revised by another experienced researcher; later, their opinions were discussed until a final consensus. As a result, three CSDs were produced.

From the CSD construction, tables resulted from this analysis were carefully read. During this process, meaningful elements of each speech were highlighted and reflections upon them were made separately. Subsequently, similar elements or those that referred to the same category were organized in interpretative axes. These were not predetermined, but rather named according to their meaning; they will be presented in the Section “Discussion” of this article.

### Quantitative Data Collection and Analysis

Prior to and after the intervention (i.e., Pre and Post), participants were evaluated for anthropometric and body composition assessments (total BW, FM, FFM, and waist and hip circumferences) and for psychological and behavioral assessments using self-applied questionnaires and scales that aimed to evaluate binge eating behaviors, disordered eating attitudes, body perception, and dissatisfaction and attitudes toward the body.

A maximal graded exercise test was performed in a treadmill (Centurion 200, Micromed, Brazil), with increments in velocity and grade at every minute until volitional exhaustion, as previously described elsewhere (24). Oxygen consumption and carbon dioxide output were obtained through breath-by-breath sampling and expressed as a 30-s average using an indirect calorimetric system (Cortex – model Metalyzer IIIB, Leipzig, Germany). Peak oxygen uptake was considered as the average of the final 30 s of the test.

Body weight was measured with an electronic-load scale in kilograms, to the nearest 0.1 kg with the participants wearing light clothing. Height was measured with a fixed stadiometer in centimeter, to the nearest 0.1 cm. Weight and height assessments were used for the BMI calculation [BW (kg)/height (m)^2^].

Waist circumference was measured with a flexible tape at the smaller circumference between the superior border of the iliac crest and the lowest rib margin in cm, to the nearest 1 cm. Hip circumference was also measured with a flexible tape at the level of the iliac crest in centimeter, to the nearest 1 cm.

Total FM and FFM were assessed using hydrostatic weighing as previously described (25). Briefly, each participant was weighed underwater at least eight times after maximum expiration; the mean of the three highest values was considered the underwater weight. Body density was determined according to Wilmore and Behnke (26), body fat according to Siri (27), and residual volume according to Goldman and Becklake (28).

To evaluate participants’ eating attitudes (defined as thoughts, feelings, beliefs, and behaviors toward eating) (29), the Disordered Eating Attitude Scale – DEAS – was used (30). This scale comprises 25 questions divided into 5 subscales including: relationship with food (e.g., I am angry when I feel hungry; I am afraid to start eating and not be able to stop); concerns about eating and BW gain (e.g., I worry about how much a certain kind of food or meal will make me gain weight); restrictive and compensatory practices (e.g., Do you “skip” meals to avoid putting on weight?); feelings toward eating (e.g., Do you feel pleasure when you eat? Does eating ever feel unnatural to you?); and idea of normal eating (e.g., How healthy and necessary do you consider consumption of breads, rice, and pasta? Do you believe that it is normal to eat sometimes just because you are sad, upset, or bored?). Higher scores mean more dysfunctional attitudes. To evaluate participants’ binge eating behavior, the Binge Eating Scale – BES was used (31). This scale comprises 16 items and 62 affirmations. For each item, the participant has to choose the one affirmation that best represents her answer. Each affirmation has a correspondent score from 0 to 3 that corresponds, respectively, to the absence and maximum gravity of the binge eating behavior. The final score is the resultant sum of points of each item. Participants were classified according to the following scores: scores ≤17 classify the behavior as absent; scores between 18 and 26 classify the behavior as moderate, and scores ≥than 27 classify it as severe.

To assess the attitudes that participants hold toward their bodies, the Body Attitude Questionnaire (BAQ) was used. The BAQ consists of a 44-item self-administered questionnaire whose subscales encompass six distinct aspects of body experience: feeling fat, body disparagement, strength and fitness, salience of weight and shape, attractiveness, and lower body fatness. Participants responded to statements using a five-point scale that ranged from “strongly disagree” to “strongly agree.” In all assessed attitudes, higher scores indicate a greater importance of the body attitude [developed by Ben-Tovim and Walker (32); validated in Brazil by Scagliusi et al. (33)]. Body perception and dissatisfaction were evaluated by the Figure Rating Scale [developed by Stunkard et al. (34); validated in Brazil by Scagliusi et al. (35)]. It consists of nine female figures numbered 1–9, ranging from thin to obese. Participants had to choose the figure they believed best represented their current body (whose number corresponds to the “body perception” variable) and a figure they believed represented their ideal body. The body dissatisfaction score was calculated by subtracting the number of the ideal figure from the number of the current figure. Higher body dissatisfaction scores indicate the desire to decrease body size.

The data are presented as means and SD, percent change, 95% CI of the differences, and effect sizes (ES), except when otherwise stated. The significance level was set at *p* ≤ 0.05. The dependent variables were compared using the Students *t*-test for dependent samples, using SAS version 8.2.

## Results

Of the 30 participants who enrolled in the intervention, 16 dropped out (3 moved to another city, 3 did not have time availability, 1 became pregnant, 4 for personal reasons, and 5 for health reasons). Figure 2 illustrates the initial number of participants and the following attritions.

Table 1 shows the socio-demographic characteristics of the participants.

**Table 1:** **General characteristics of women participating of a non-prescriptive multidisciplinary intervention based on the Health at Every Size^®^ philosophy (*n* = 14)**.

Characteristic	Mean	%
Age (years)	4.5 (7.1)	
Peak oxygen uptake (mL/kg/min)	34.3 (7.5)	
**Education**		
Graduated from high school		28.6
Graduated from college		71.4
**Weight gain history**		
Childhood		35.7
Adolescence		7.1
Adulthood		57.2
**Occupation**		
Teacher		21.4
Housewife or retired		28.6
Student		7.1
Autonomous		14.3
Public employee		7.2
Outsourced employee		21.4
**Relationship status**		
Single		35.7
Married		42.9
Divorced		14.3
Widowed		7.1

*Data are expressed as means (SD), and percentages (%)*.

### Quantitative Data

Anthropometric and body composition assessments are depicted in Table 2. Although FFM was increased and waist and hip circumferences were slightly decreased, these changes did not reach statistical significance. Regarding age, weight, and BMI, no statistically significant differences were observed among participants who completed or dropped out of the study.

**Table 2:** **Anthropometric and body composition assessments before and after the intervention**.

Variable	Pre (*n* = 14)	Post (*n* = 14)	Change values (%)	CI (95%)	ES	*p* Value
Body weight (kg)*	96.9 (16.4)	93.4 (17.9)	−3.6	1.40 to 5.16	−0.21	0.003
Body mass Index (kg/m^2^)*	37.0 (5.7)	35.8 (6.4)	−3.2	0.55 to 1.90	−0.22	0.003
Body fat mass (kg)*	42.2 (10.5)	36.7 (13.6)	−13.0	2.59 to 7.72	−0.50	0.001
Body fat mass (%)*	43.2 (4.5)	38.4 (8.1)	−11.1	1.63 to 7.39	−0.99	0.01
Body fat-free mass (%)	54.7 (7.7)	56.8 (8.1)	+3.8	−4.77 to 0.63	+0.25	0.27
Waist circumference (cm)	112.6 (12.3)	110.4 (12.4)	−2.0	−0.06 to 4.09	−0.16	0.10
Hip circumference (cm)	127.4 (14.6)	122.3 (14.3)	−4.0	0.35 to 11.9	−0.37	0.10

*Data are expressed as means (SD), percent change, 95% confidence interval (CI) of the differences, and effect sizes (ES). Paired Student’s *T* tests were used to assess possible differences between time points*.

*Indicate which findings are statistically significant. Significance defined as *p* ≤ 0.05.

Data regarding eating behavior and body image are depicted in Table 3. Regarding the BAQ scale, subscales “Lower body fatness” and “Strength and fitness” reached statistical significance. Participants exhibited less dysfunctional attitudes toward eating but only subscale “Idea of normal eating” reached statistical significance. A trend toward a decrease was observed in the subscale “Feelings toward eating.” Notably, regarding the binge eating behavior, at Pre, 35.7% of the participants were classified as absent of the behavior and 57.1% of the participants were classified with a moderate behavior. At Post, 78.6% of the participants were classified as absent of the behavior and 14.3% of the participants were classified with a moderate behavior. No differences were observed in the severe classification of the behavior during the intervention (7.1%). Finally, regarding the Figure Rating Scale, subscales “Current body size” and “Current body size-Ideal body size” reached statistical significance.

**Table 3:** **Psychological and behavioral assessments before and after the intervention**.

Variable	Pre (*n* = 14)	Post (*n* = 14)	CI (95%)	*p* Value
Binge Eating Scale*	18.6 (6.0)	13.1 (7.7)	1.78 to 9.42	0.014
Disordered Eating Attitude Scale				
* Subscale 1 – Relationship with food*	25.4 (6.4)	23.4 (8.6)	−1.0 to 5.64	0.28
* Subscale 2 – Concerns about eating and weight gain*	7.3 (2.2)	7.3 (2.3)	−1.28 to 1.35	0.92
* Subscale 3 – Restrictive and compensatory practices*	7.3 (3.6)	5.6 (2.1)	0.14 to 3.71	0.10
* Subscale 4 – Feelings toward eating*	4.1 (1.8)	3.3 (1.1)	0.28 to 1.71	0.08
Subscale 5 – Idea of normal eating*	23.1 (6.7)	28.8 (7.6)	−9.71 to −2.14	0.017
* Total Score*	67.3 (13.1)	68.4 (14.7)	−6.78 to 5.63	0.77
Body Attitude Questionnaire				
* Subscale 1 – Attractiveness*	15.4 (3.7)	16.2 (3.0)	−1.85 to 0.14	0.14
* Subscale 2 – Body disparagement*	20.1 (6.2)	19.4 (6.7)	−1.06 to 3.64	0.51
* Subscale 3 – Feeling fat*	50.4 (8.0)	48.0 (7.1)	−0.21 to 5.28	0.12
* Subscale 4 – Salience of weight and shape*	25.5 (4.0)	25.1 (2.9)	−1.64 to 2.43	0.74
Subscale 5 – Lower body fatness*	15.9 (2.6)	14.4 (2.0)	0.14 to 2.78	0.05
Subscale 6 – Strength and fitness*	16.9 (3.7)	18.4 (4.1)	−2.64 to −0.21	0.05
Figure Rating Scale				
Subscale 1 – Current body size*	6.9 (1.0)	6.1 (1.1)	0.42 to 1.0	0.001
* Subscale 2 – Ideal body size*	4.1 (0.8)	3.9 (1.1)	−0.21 to 0.5	0.5
Subscale 3 – Current body size – Ideal body size*	2.8 (0.8)	2.2 (1.0)	0.07 to 1.07	0.05

*Data are expressed as means (SD), percent change, and 95% confidence interval (CI) of the differences. Paired Student’s *T* tests were used to assess possible differences between time points*.

*Indicate which findings are statistically significant. Significance defined as *p* ≤ 0.05.

### Qualitative Data

Tables presenting the CI and their CSD regarding the general experiences of participants in the non-prescriptive multidisciplinary program based on the HAES^®^ philosophy are depicted in the Supplementary Material. Four interpretative axes constructed from the CSD arising from the focus groups were used to discuss the results and are presented in the section “Discussion”. The interpretative axes were named as the following: the intervention as a period of discoveries; shifting parameters: psychological, physical, and behavioral changes; eating changes, and; redefining success. Quotes from the participants’ discourses are also used to illustrate key points and the corresponding identification can be found on the tables presented in the Supplementary Material.

## Discussion

This study sought to explore – qualitatively and quantitatively – the effects of a non-prescriptive multi-disciplinary program based on the HAES^®^ philosophy. The qualitative method allowed obtaining detailed data from the participants’ perspective and the quantitative method allowed evaluating potential changes in their body composition and psychological and behavioral assessments. To the best of our knowledge, this is the first study to use a mixed-method approach to access the quantitative and qualitative effects of a HAES^®^-based program on these parameters and on the meaning of these parameters to the participants.

### The Intervention as a Period of Discoveries

Despite its negative consequences, people can still feel very tempted to diet. Diets are highly advertised and can be very appealing, since they not only promise weight loss *per se*, but also a “better life,” and professional and relationship success (36). For instance, Barberia et al. (37) qualitatively explored beliefs and socio-cognitive factors in a group of obese and overweight Spanish women undertaking a weight-loss treatment. The participants believed that dieting would make them lose weight and this was a highly valued outcome since they believed that this would improve their health and physical appearance. One participant, for example, said that she wanted to lose weight because she would feel less tired, which suggests that she believed that by dieting she would improve her quality of life.

In our intervention, a broader approach was proposed: rather than focusing on the BW and what to do about it, participants were encouraged to reflect whether they had struggled with body and eating issues throughout their lives and whether the diets experiences intensified these aspects. More specifically, they were stimulated to gain new perspectives and to develop skills of their own that would enable them to change their eating behaviors (if and when necessary), their level of physical activity, and patterns of thinking and acting that might had been preventing them to change.

As a result, our participants reported that the intervention was a period of discoveries, as exemplified by the following statement – *“the intervention met an enormous desire to change* [.] *it’s being a rediscovery”* (CI 1A). Another statement complemented that such aspect when they said that they felt impelled to restart past activities (CI 1E). Interestingly, our participants highlighted a shift in the expectations regarding their bodies: initially, they reported an expectation that by losing weight they would become a *“very sexy woman”* (CI 2C), but after the intervention they realized that such change might not be achievable and reported feeling positive about it (CI 2C). It is worth noticing that, differently from what was reported by the participants of Barberia et al. (37), in which the weight loss seemed the only mean to change the health and physical appearance of the participants, the discourses abovementioned brought up different perceptions, suggesting that the weight loss was not mandatory for our participants to start new activities, as exemplified in CI 1A – *I’m going after things that I like and I think the intervention has been given me strength in this sense.”* Moreover, weight loss was not mandatory for them to develop a better perception and attitude toward their bodies. It is likely that if they kept their previous weight-centered way of thinking they would feel disappointed and would possibly abandon the intervention for not meeting their goals.

The qualitative results indicate even more complex effects. It can be suggested, for example, that the benefits reported impacted on the participants’ willingness to include extra physical activities in their routine (*“I’m taking part in a marathon now, I said ‘wow, it’s hard, but it’s better than before, this competition is easier than the other’”* – CI 1C, complemented by CI 4A) and to continue with the intervention, as suggested on the discourse in which they said that even if they could not arrive to the activities, they would come *“just to say hi”* (CI 1B). It seems that supporting them to discover what they could do despite their BW resulted in more meaningful gains rather than focusing on what is usually just a mean to an end in weigh-centered approaches: the weight loss. CI 1E illustrates that idea – *“happiness is not in the weight loss, it is about feeling good. So if my ‘feel good’ today is coming to college, doing my things, it may be part of my happiness.”* Additionally, the participants reported feeling *“more empowered to face some challenges”* (CI 4C). These results are of great value as they further support the effectiveness of this HAES^®^-based program not only in biological, but also in psychological and sociocultural parameters.

Thus, one may suggest that other variables seem to reflect more significant health benefits than only the weight loss *per se*, and therefore, should be of high value to indicate the success of interventions for the treatment and prevention of obesity. A wider discussion regarding this will be explored in the interpretative axe 4.2.

### Shifting Parameters: Psychological, Physical, and Behavioral Changes

The dominant view in the obesity discourse considers that weight loss *per se* is a synonymous for improved health. As Aphramor (38) discusses, this discourse is sustained by the belief that weight loss is always possible. This assumption hardly considers other clinical outcomes that are important to health improvements and usually overlooks the processes by which the weight loss may be achieved (e.g., at the expense of health). Moreover, a number of studies do not support that either the weight loss or the health benefits are sustained after weight-centered programs. Pamuk et al. (39) examined the relationship between maximum weight, weight loss, and mortality in 2,140 males and 2,550 females (aged 45–74 years). After adjusting for variables such as age, ethnicity, smoking, pre-existing diseases, and BMI, for those with a BMI between 26 and 29 kg/m^2^, the risk of mortality increased with a greater weight loss. The participants who lost 15% or more of their maximum weight had a twofold increase in the mortality risk in comparison with those who lost <5%. For women with a BMI ≥29 kg/m^2^, the risk of mortality increased proportionally to the amount of weight lost.

We observed a modest albeit significant weight loss, which indicates that this kind of intervention: (1) does not cause weight gain, as some critics of the HAES^®^ have previously suggested and; (2) in a not weight-centered intervention, a modest weight loss may occur as a consequence of new behaviors. Interestingly, our results showed a greater loss of body fat (in kilogram or in percentage) than BW, highlighting the further benefits of physical activity practice on body composition (40). Moreover, although not statistically significant, decreases in the hip and waist circumferences were also observed. Critics of the HAES^®^ express concern that its principles (e.g., encouraging body acceptance, not prescribing a diet) will lead individuals to make poor nutritional choices and will lead a state of passivity or inaction, resulting in weight gain. An expressive body of evidence has disproven these concerns. Therefore, the abovementioned body assessments taken in our study aimed to add evidence to the HAES^®^-based interventions regarding this matter, showing that our intervention had, as well, not resulted in weight gain. Also, our participants had autonomy to decide whether they wanted or not to know the result of their body assessments, and that these data were not used to motivate them to achieve a given weight. It was in accordance with the HAES^®^ philosophy once that it assumes that “the movement toward a healthier lifestyle will, over time for most people, produce a weight that is healthy for that person” (14). It does not prohibit weight changes, but provides strategies that have as major principle the promotion of physical and mental health independent of whether weight loss is a consequence or not of this process.

Finally, although our participants had a modest weight loss, they seemed to have rediscovered other possibilities for their bodies, especially during the practice of joyful physical activities, which may have contributed to the increase in the “strength and fitness” score in the BAQ. The participants’ report on CI 1C reinforces this idea – *“Today, every time I come here is by walking from the train station* [.]*. And I thought it was very nice because I was not used to walk towards the subway station.”* The increase in their physical fitness seemed to have triggered their willingness to include a range of other activities in their routines, as expressed in CI 4A when they said that they pictured themselves *“going out more, enjoying more of what the city has to offer,”* which that did not seem to be a possibility before the intervention. Additionally, our participants provided “cues” that they perceived and related to their bodies in a different way. First, they seemed not to think about the weight loss as much as before, suggested by the following discourse –*“it’s about maintaining the exercise* [.] *consequently we’ll lose weight, but it’s not the goal”* (CI 4A). Also, the decreases in the Figure Rating Scale subscales “Current body size” (which is a measure of body perception) and “Current body size-Ideal body size” (which is a measure of body dissatisfaction) indicate that our participants developed a better perception of their bodies and were more comfortable and less dissatisfied with their current physical condition. CI 2C illustrates that – *“I thought I would lose weight and I would become a blonde bombshell. And I found it very interesting because I had this imaginary and I think that was not nice, this isn’t the right way.”* The diminishments in the abovementioned scales, in addition to the decrease in the “lower body fatness” score in the BAQ, suggest that, despite not having an expressive weight loss, our participants developed a better body image. CI 4B and 4C indicated that some participants still held an expectation regarding a future weight loss. It is likely that some of them needed more time to replace this expectation with a new paradigm. Nevertheless, these CIs seemed to reflect a more realistic expectation and indicate movements toward a healthier lifestyle, what may, over time, produce a weight that is healthy for them, being in accordance with what one of the HAES^®^ principles assume (14).

### Eating Changes

In this nutritional intervention, diets were not prescribed. More importantly than the nutritional therapists helping the participants to plan which and the amount of food that would be more appropriate for them to consume, these professionals helped them to build a solid and detailed pathway of how to make the proposed changes feasible, which would, in time, enable them to change their eating behaviors (if and when necessary).

Leblanc et al. (15) evaluated the effects of a HAES^®^-based intervention on the consumption and food intake of 144 overweight/obese women (mean age of 42.3 ± 5.6 years). They were randomly assigned to one of the following groups: HAES^®^, social support, or control group. The participants filled in a 3-day food record and the Three-Factor Eating Questionnaire in order to evaluate their eating behaviors. The authors observed that the food intake and the frequency of the “nibbling” (i.e., to eat something by taking a lot of small bites) decreased in the three groups, while the proportion of energy from breakfast increased. In the HAES^®^ group, the increased perception of the hunger and satiety cues was related with a diminishment of the total of the daily energy consumed.

In our study, the food record was also used; it consisted of a daily register of what the participants ate and also included notes of the time and duration of the meal, feelings, and thoughts related to food and the satiety and hunger perception. Our participants reported that being more aware of the hunger and satiety cues helped them to reduce their food quantity (CI 2D), as observed in the study abovementioned. A similar result can be seen in the study of Provencher et al. (17). The researchers assessed the effects of a 4-month HAES^®^ intervention on numerous variables, including the appetite sensations. It was observed that participants from the HAES^®^ group showed a significant decrease over time in their scores of susceptibility to hunger (simple effect of time; *p* = 0.0001), while that such decrease was not observed in the control group. Our participants also seemed to have benefited when certain patterns of behaviors were pointed to them, as follows: it was declared that they had not realized that food was the way they had to manage certain situations. By being aware of this, they appeared to have gained skills to deal with this situations properly, without constantly turning to food, as stated in the following discourses – *to to be able to realize and say ‘I ate more than I should and it wasn’t because I was hungry, it was because of other situation and I need to notice myself not to do it again’”* (CI 2B) and CI 4B. Other strategy used by the nutritional therapists was to help participants to neutralize food, that is, not to classify food dichotomously. Apparently, it was effective to help the participants to refrain from the restrictive behavior (when it was the case) – *“it’s like this, you can restrain yourself today, but tomorrow is another story”* (CI 2E), also complemented by CI 4B – *“Today I know that I can eat a certain amount and it’s up to me knowing when to stop.”* This was also reinforced when they said that they could manage what they enjoyed to eat – *“you will never stop eating something that is pleasant to you”* (CI 2E), suggesting that they did not restrain their eating anymore. In accordance with these data, the BES scores showed important changes regarding eating behaviors as well. At the end of the intervention, the mean value of this scale revealed a score lower than 17, which classifies the binge eating behavior as absent. Although it is not possible to affirm that our participants had the binge eating disorder at baseline, it can be suggested that the tools applied in our intervention were effective to help them to improve their eating behavior. Finally, our participants also reported that by planning their meals (specifically involving consumption of small, frequent meals) they realized that they felt less hungry and consequently ate less than they did before (CI 2E), suggesting that this positive perception impelled them to continue with this change – *“I stay a long time without eating and when I eat I eat a lot”* (CI 2E).

People who are constantly on a diet may feel that nutritional changes are very hard, demanding and not truly achievable. From the discourses highlighted above, it seems that the nutritional therapists could effectively help the participants to make eating changes. Our participants seemed to have made positive changes and were also confident and skilled to maintain these changes in spite of the intervention conclusion (CI 4B), differently from what might have happened if diets were prescribed. Furthermore, the discourses highlighted in this interpretative axis also suggest that the nutritional intervention helped the participants to understand the plural roles of food (e.g., comfort, pleasure, celebration, care) rather than just a way to “fulfill the body biologically.” The decrease in the DEAS subscale “Feelings toward eating” (in which higher scores indicate less food memories and less pleasure with eating), reinforces this suggestion. The perception and acceptance of the food plurality can be of great value for the maintenance of the positive changes in the eating behavior accomplished by the participants.

### Redefining Success

It was suggested from the discourses that our participants successfully changed some past assumptions. For instance, in CI 2A and 1F, they highlighted that they could realize limitations and that the process of changes is a lifetime ongoing process. They seemed to have changed their dichotomous way of thinking – in which changes would either happen suddenly or would never happen. It appears that by realizing that changes are part of a permanent process they could shift from a discouraged to an encouraged posture to make changes, like including exercises in their lives and realizing they could lose weight (CI 1C and 2D, respectively) which did not seem a possibility in the past. This perception seemed to have helped them to feel empowered to make further and more complex actions.

Lasikiewicz et al. (41) conducted a review to assess the psychological consequences of interventions that aimed at behavioral changes or at weight loss (with or without physical activity) of obese people. It was observed that the weight loss was positively associated with certain psychological improvements, related, for example, with body image and quality of life. However, the authors also pointed out that in studies that offered a greater social support and used strategies to increase the self-acceptance of the participants, the absence of changes in BW did not interfere in the psychological improvements observed. It was not under the scope of this study to evaluate psychological improvements of the participants. However, it was possible to assess some interesting aspects by asking what our participants regarded as a success of the treatment and what it meant to them. In their answers, the followings aspects were highlighted: first, success was to be able to reflect about issues and to realize limitations – *“to me it’s already a success, take from the program the perception that you can realize, you can know you can do more. And also realize our limitations”* (CI 3A). Our participants also talked about the intervention as a broader treatment, suggesting that it was more than just a nutritional, philosophical, and an exercise program (CI 3A); they also reported that the intervention was a place where they felt welcomed and accepted and where they could relate and identify with their colleagues (CI 1B and 3A). A second aspect regarded as a success was to be able to give continuity to what they have learned (CI 3B).

Polivy and Herman (36) investigated the reasons why people tend to fail in their self-change attempts and examined how they interpret and what are the consequences of such failures. The authors argue that people’s expectation tend to exceed what is feasible and lead them to reject more modest goals; also, people usually believe they will change more quickly and easily than is possible. Polivy and Herman (36) also discuss that people who believe that they can succeed are more likely to actually succeed than people who do not. In the discourses abovementioned, our participants seemed to have understood their possibilities and potential to change. They reported having realized that changes are possible, but are part of a process, suggesting that they were able to accept more modest, but achievable goals. This perception appeared to have reflected in their confidence, suggesting that they acquired self-efficacy and are more likely to make efforts and to persist when facing obstacles without the professional support. Our participants reported being confident about what they should do, suggesting their acquired skills to deal with different situations autonomously, as suggested by the following statements: *“I see myself putting into practice what I have learned”* (CI 4A) and *“from now on, being able to lose weight and feeling well depend on me”* (CI 4B).

Further studies are necessary to confirm such assumptions. It would be interesting to analyze whether self-efficacy is indeed important to a more lasting and significant improvement. Qualitative inquiries applying individual interviews could address such aspect.

### Study Limitations

Among the limitations of this study, we highlight the dropout of participants. In a 6-month dietary intervention that aimed at weight loss of its obese participants, Colombo et al. (42) reported a 56% dropout until the completion of the study. The authors argued that factors such as the age at which people started to diet and the amount of BW lost at the beginning of the given intervention were potentially relevant to the dropouts. Also, some characteristics of the participants, such as a hostile behavior, also appeared to contribute to such dropout rate (42). In accordance, in our 1-year study, 53.3% of the original sample dropped out the intervention before its conclusion. However, the final sample allowed an in-depth data collection, a crucial aspect of qualitative inquiries, enabling the study’s objective to be accomplished. Moreover, differently from what was stated in the study abovementioned (42), the reasons provided for the dropouts were external to the intervention. We highlight the high attrition in light of the study’s avoidance of weight-related outcomes. Also, this attrition may be reflection of the availability the participants needed to dispend to participate in our intervention. Nonetheless, this attrition is similar to attrition rates seen in traditional behavioral weight-loss trials that focus on actual weight loss in addition to adopting healthier lifestyles and attitudes. The participants who remained likely skew positively in regards to psychological and behavioral outcomes measured in this study. Regarding the collective characteristic of the focus group, some ideas might have been left unmentioned by participants. However, according to the observer notes, our participants were expressively engaged in the discussions and expressed different ideas, indicating that they felt welcomed to express their opinion. Regarding the quantitative analysis, despite the small sample size, it was possible to observe statistically significant changes in some parameters. Importantly, these data were complementary to the qualitative findings, having both methods gained strength when combined. Also, to the best of our knowledge, this was the first mixed-method study, which had such proposal explored in the area. Another limitation is related to the absence of a control group, preventing the evaluation of causality. However, it was a pilot study and we intend to overcome this limitation in a next intervention. We also highlight the absence of an evaluation of the participants who dropped out from the intervention, as well as the absence of a follow-up.

## Conclusion

The intervention was reported to motivate the participants to engage in novel activities and to help them to have a more realistic expectation regarding BW. This result was accompanied by modest but significant improvements in body composition, psychological and behavioral quantitative assessments, indicating that our participants were more comfortable with their physical characteristics. The slight increase in their FFM indicates that the participants could retain lean body mass while losing FM. Eating-wise, participants reported having benefited from the hunger and satiety cues and from the consumption of more frequent meals. They declared being able to relate feelings with eating choices, to refrain from the restrained behavior, and seemed to have developed a more functional attitude regard eating. Finally, participants declared realizing that the process of changes was ongoing.

This study further supports that the HAES^®^ is an innovative approach, giving people opportunity to change the way they relate with their bodies and food. If only quantitative analyses had been conducted, the indisputable positive psychosocial and physiological effects of this intervention may have been undervalued. Our findings demonstrate the high value of this type of study and encourage the development of randomized controlled trials to confirm the efficacy of such intervention.

## Author Contributions

MU, BG, FB, RU, and FS conceived the study, analyzed the findings, and wrote the article. RU was the focus groups moderator, PS was the co-moderator of the focus groups, and both contributed to the interpretation of the study. All authors read, reviewed the manuscript critically, and approved the final version.

## Conflict of Interest Statement

The authors declare that the research was conducted in the absence of any commercial or financial relationships that could be construed as a potential conflict of interest.

## References

[1] Leske S Strodl E Hou X-Y A qualitative study of the determinants of dieting and non-dieting approaches in overweight/obese Australian adults. BMC Public Health (2012) 12(1):1086 10.1186/1471-2458-12-1086 23249115 PMC3541951

[2] Instituto Brasileiro de Geografia e Estatística. Antropometria e estado nutricional de crianças, adolescentes e adultos no Brasil: Pesquisa de Orçamentos Familiares 2008-2009. Rio de Janeiro (2010).

[3] Gagnon-Girouard MP Provencher CBG Tremblay A Mongeau L Boivin S Lemieux S Psychological impact of a ‘Health-at-Every-Size’ intervention on weight-preoccupied overweight/obese women. J Obes (2010) 2010:1–12. 10.1155/2010/928097 PMC2925467 20798861

[4] O’Hara L Gregg J. The war on obesity: a social determinant of health. Health Promot J Austr (2006) 17(3):260–3. 17176244 10.1071/he06260

[5] Douketis JD Morrison KM Hramiak IM Sharma AM Canadian clinical practice guidelines on the management and prevention of obesity in adults and children [summary]. Can Med Assoc J (2007) 176(8):1–13. 10.1503/cmaj.061409 PMC1839777 17420481

[6] Barte JCM Veldwijk J Teixeira PJ Sacks FM Bemelmans WJE. Differences in weight loss across different BMI classes: a meta-analysis of the effects of interventions with diet and exercise. Int J Behav Med (2014) 21:784–93. 10.1007/s12529-013-9355-5 24797705

[7] Johnston BC Kanters S Bandayrel K Wu P Naji F Siemieniuk RA Comparison of weight loss among named diet programs in overweight and obese adults – a meta-analysis. JAMA (2014) 312(9):923–33. 10.1001/jama.2014.10397 25182101

[8] Verhoef SPM Camps SGJ Bouwman FG Mariman ECM Westerterp KR. Physiological response of adipocytes to weight loss and maintenance. PLoS One (2013) 8(3):e58011. 10.1371/journal.pone.0058011 23505452 PMC3591449

[9] Have M Beaufort ID Teixeira PJ Mackenbach JP van der Heide A. Ethics and prevention of overweight and obesity: an inventory. Int J Obes (2011) 12(9):669–79. 10.1111/j.1467-789X.2011.00880.x 21545391

[10] Bacon L Aphramor L. Weight science: evaluating the evidence for a paradigm shift. Nutr J (2011) 10(1):1–13. 10.1186/1475-2891-10-9 21261939 PMC3041737

[11] Ochner CN Barrios DM Lee CD Pi-Sunyer FX. Biological mechanisms that promote weight regain following weight loss in obese humans. Physiol Behav (2013) 120:106–13. 10.1016/j.physbeh.2013.07.009 23911805 PMC3797148

[12] Tylka TL Annunziato RA Burgard D Daníelsdóttir S Shuman E Davis C The weight-inclusive versus weight-normative approach to health: evaluating the evidence for prioritizing well-being over weight loss. J Obes (2014) 2014:983435. 10.1155/2014/983495 25147734 PMC4132299

[13] Association for Size Diversity and Health. (2003). Available from: https://www.sizediversityandhealth.org/

[14] Kratina K King NL Hayes D Moving Away From Diets: Healing Eating Problems and Exercise Resistance. 2nd ed Helm Publishing (2003).

[15] Leblanc V Provencher V Bégin C Corneau L Tremblay A Lemieux S Impact of a health at every size intervention on changes in dietary intakes and eating patterns in premenopausal overweight women: results of a randomized trial. Clin Nutr (2012) 31(4):481–8. 10.1016/j.clnu.2011.12.013 22296874

[16] Bacon L Stern JS Van Loan MD Nancy L Keim NL. Size acceptance and intuitive eating improve health for obese, female chronic dieters. J Am Diet Assoc (2005) 105(6):929–36. 10.1016/j.jada.2005.03.011 15942543

[17] Provencher V Bégin C Tremblay A Mongeau L Corneau L Dodin S Health at every size and eating behaviors: 1-year follow-up results of a size acceptance intervention. J Am Diet Assoc (2009) 109:1854–61. 10.1016/j.jada.2009.08.017 19857626

[18] Katzer L Bradshaw AJ Horwath CC Gray AR O’Brien S Joyce J Evaluation of a ‘nondieting’ stress reduction program for overweight women: a randomized trial. Am J Health Promot (2008) 22(4):264–74. 10.4278/060728113R1.1 18421891

[19] Borràs V López P Lozares C La articulación entre lo cuantitativo y lo cualitativo: de las grandes encuestas a la recogida de datos intensive. QUESTIIÓ (1999) 23(3):525–41.

[20] Spahn JM Reeves RS Keim KS Laquatra I Kellogg M Jortberg B State of the evidence regarding behavior change theories and strategies in nutrition counseling to facilitate health and food behavior change. J Am Diet Assoc (2010) 110(6):879–91. 10.1016/j.jada.2010.03.021 20497777

[21] Liamputtong P Focus Group Methodology: Principle and Practice. London: SAGE (2011).

[22] Ulian MD Gualano B Benatti FB de Campos-Ferraz PL Roble OJ Modesto BT “Now I Can Do Better”: a study of obese women’s experiences following a non-prescriptive nutritional intervention. Clin Med Insights Womens Health (2015) 8:13–24. 10.4137/CMWH.S23163 26417206 PMC4573064

[23] Lefévre F Lefévre AMC O discurso do sujeito coletivo: Um novo enfoque em pesquisa qualitativa (Desdobramentos). 2nd ed Caxias do Sul: Educs (2005).

[24] Prado DM Benatti FB de Sá-Pinto AL Hayashi AP Gualano B Pereira RM Exercise training in childhood-onset systemic lupus erythematosus: a controlled randomized trial. Arthritis Res Ther (2013) 15(2):R46. 10.1186/ar4205 23531226 PMC3672722

[25] Artioli GG Iglesias RT Franchini E Gualano B Kashiwagura DB Solis MY Rapid weight loss followed by recovery time does not affect judo-related performance. J Sports Sci (2010) 28(1):21–32. 10.1080/02640410903428574 20035492

[26] Wilmore JH Behnke AR An anthropometric estimation of body density and lean body weight in young men. J Appl Physiol (1969) 27(1):25–31. 5786965 10.1152/jappl.1969.27.1.25

[27] Siri WE Body composition from fluid space and density. In: Brozek J Hanschel A, editors. Techniques for Measuring Body Composition. Washington, DC: National Academy of Science (1961). p. 223–44.

[28] Goldman HI Becklake MR Respiratory function tests; normal values at median altitudes and the prediction of normal results. Am Rev Respir Dis (1959) 79(4):457–67. 10.1164/artpd.1959.79.4.457 13650117

[29] Alvarenga M Scagliusi FB Philippi ST Nutrição e transtornos alimentares: avaliação e tratamento. Barueri: Manole (2011).

[30] Alvarenga MS Pereira RF Scagliusi FB Philippi ST Estima CCP Croll J Psychometric evaluation of the Disordered Eating Attitude Scale (DEAS). English version. Appetite (2010) 55(2):374–6. 10.1016/j.appet.2010.07.003 20624435

[31] Freitas S Lopes CS Coutinho W Appolinario JC Translation and adaptation into Portuguese of the Binge-Eating Scale. Rev Bras Psiquiat (2001) 23(4):215–20. 10.1590/S1516-44462001000400008

[32] Ben-Tovim D Walker MK. The development of the Ben-Tovim Walker body attitudes questionnaire (BAQ), a new measure of women’s attitudes towards their own bodies. Psychol Med (1991) 21:775–84. 10.1017/S0033291700022406 1946865

[33] Scagliusi FB Polacow VO Cordás TA Coelho D Alvarenga MS Philippi ST Psychometric testing and applications of the Body Attitudes Questionnaire translated into Portuguese. Percept Motor Skill (2005) 101(1):25–41. 10.2466/pms.101.1.25-41 16350606

[34] Stunkard AJ Sorenson T Schulsinger F Use of Adaption Registry for the study of obesity and thinness. In: Kety S Rowland L Sidman R editors. The Genetics of Neurological and Psychiatric Disorders. New York, NY: Raven Press (1983). p. 115–20.

[35] Scagliusi FB Alvarenga M Polacow VO Cordas T de Oliveira GQ Coelho D Concurrent and discriminant validity of the Stunkard’s figure rating scale adapted into Portuguese. Appetite (2006) 47(1):77–82. 10.1016/j.appet.2006.02.010 16750589

[36] Polivy J Herman CP. If at first you don’t succeed: false hopes of self-change. Am Psychol (2002) 57(9):677–89. 10.1037/0003-066X.57.9.677 12237978

[37] Barberia AM Attree M Todd C Understanding eating behaviors in Spanish women enrolled in a weight-loss treatment. J Clin Nurs (2008) 17(7):957–66. 10.1111/j.1365-2702.2007.02073.x 18321293

[38] Aphramor L. Validity of claims made in weight management research: a narrative review of dietetic articles. Nutr J (2010) 9:30. 10.1186/1475-2891-9-30 20646282 PMC2916886

[39] Pamuk ER Williamson DF Madans J Serdula MK Kleinman JC Byers T Weight loss and mortality in a national cohort of adults, 1971–1987. Am J Epidemiol (1992) 136(6):686–97. 1442735 10.1093/oxfordjournals.aje.a116548

[40] Votruba SB Horvitz MA Schoeller DA The role of exercise in the treatment of obesity. Nutrition (2000) 16(3):179–88. 10.1016/S0899-9007(99)00264-6 10705072

[41] Lasikiewicz N Myrissa K Hoyland A Lawton CL Psychological benefits of weight loss following behavioral and/or dietary weight loss interventions. A systematic research review. Appetite (2014) 72:123–37. 10.1016/j.appet.2013.09.017 24075862

[42] Colombo O Ferretti VV Ferraris C Trentani C Vinai P Villani S Is drop-out from obesity treatment a predictable and preventable event? Nutr J (2014) 13(13):1–7. 10.1186/1475-2891-13-13 24490952 PMC3914843

