# Supplementary material for: The Effects of a “Health at Every Size®”-Based Approach in Obese Women: A Pilot-Trial of the “Health and Wellness in Obesity” Study
Source: Front Nutr. 2015 Oct 27;2:34. doi: 10.3389/fnut.2015.00034 (PMC4621435; doi:10.3389/fnut.2015.00034)
Supplement: Supplementary file 1 [file Table_1.DOCX]

***Supplementary Material***

**The effects of a ‘Health at Every Size®’-based approach in obese women: a pilot-trial of the ‘Health and Wellness in Obesity’ study**

Mariana Dimitrov Ulian^1^*, Fabiana B. Benatti^2^, Patricia Lopes de Campos-Ferraz^3^, Odilon J. Roble^4^, Ramiro Fernandez Unsain^5^, Priscila de Morais Sato^6^, Bruna Cristina Brito^1^, Karina Akemi Murakawa^2^, Bruno T. Modesto^2^, Luiz Aburad ^1^, Rômulo Bertuzzi^2^, Antonio H. Lancha Junior^2^, Bruno Gualano^2^, Fernanda B. Scagliusi^1^.

^1^ Department of Nutrition, Faculty of Public Health, University of Sao Paulo, Sao Paulo, SP, Brazil.

^2^ School of Physical Education and Sport, University of Sao Paulo, Sao Paulo, SP, Brazil.

^3^ Faculty of Applied Sciences, State University of Campinas, Limeira, SP, Brazil.

^4^ Faculty of Physical Education, State University of Campinas, Campinas, SP, Brazil.

^5^ Faculty of Philosophy and Letters, National University of Buenos Aires, Buenos Aires, Argentina.

^6^ Institute of Health and Society, Federal University of Sao Paulo, Santos, SP, Brazil.

***Address correspondence to**: Mariana Dimitrov Ulian, Department of Nutrition, University of Sao Paulo, Faculty of Public Health, Av. Dr. Arnaldo, 715, Sao Paulo, SP, Zip Code: 01246-904, Brazil. Phone: 55 11 3061-7755. Email: m.dimitrov@usp.br.

# Supplementary Tables

Tables 1 and 2 present the central ideas (CI) and their collective subject discourses (CSD) regarding the general experiences of participants in the non-prescriptive multidisciplinary program based on the Health at Every Size® philosophy. The former regards to the first focus group and is constructed by question one; the later regards to the third focus group and is constructed by questions two and three.

| **Table 1. Central ideas and collective subject discourse constructed from the discourses expressed in the first focus group (post 3 months), conducted with women who were participating of a non-prescriptive multidisciplinary intervention based on the Health at Every Size® philosophy.** | | | | | | | | | | | | |
| --- | --- | --- | --- | --- | --- | --- | --- | --- | --- | --- | --- | --- |
| **1) Do you realize the intervention had an impact on your lives? What changed in your routine from the beginning of the intervention to the present moment?** | | | | | | | | | | | | |
| **CENTRAL IDEA** | | | | | **COLLECTIVE SUBJECT DISCOURSE** | | | | | | | |
| 1A: The intervention is good for me; it is being a period of rediscoveries and it met my interests. | | | | | *Actually the intervention is being really good for me. I guess I’ve been like this from a long time I forgot that I had a body and I think I went through some emotional issues that disconnected me from it... And then I got a job that also made me left my personal life aside... and then I also had my family that was very important and was through many issues. Then I forgot that I existed and had many issues too. I think the intervention met an enormous desire to change, a career change, a life change, it’s being a rediscovery. I’m going after things that I like and I think the intervention has been giving me strength in this sense.* | | | | | | | |
| 1B: It feels good and is pleasurable to be here, I have fun with the activities. | | | | | *It feels good, we feel happy to leave our houses to come here, I enjoy it, I feel good. I improved a lot, my mood, everything. It’s very pleasurable. Once I got stuck in traffic, and when I arrived here the period of the activity was over, but I came just to say hi, to look at. The distance seems to have decreased because people say “wow, is the activity really at USP?”. It's a pleasure. I have fun in everything I'm doing, so here for me is being really fun as well, you know? It's therapeutic.* | | | | | | | |
| 1C: The physical strength is excellent, it improved a lot. | | | | | *First of all, the physical strength is excellent, I can walk, I’m running, I’m taking part in a marathon now, I said “wow , it's hard , but it 's better than before , this competition is easier than the other”, so I’m enjoying all of this. For me what was a positive aspect is the physical conditioning that has improved a lot. All the time in my neighborhood people called me to join the running circuit and stuff, but I never went. Then, when I decided to go, for the first time I said “I'll just walk” and I felt really uncomfortable because everybody was walking by my side, but it was so good because I could accomplish the competition, because I was doing the activities here. For the second time I also walked and it was easier to accomplish the seven kilometers. For the third time I started running, but I knew I couldn’t afford to run five kilometers, so I walked three and ran one minute and then I could finish the race. Regarding to the walk from here to the train station... At first it seemed that it was a travel… Today every time I come here is by walking from the train station. And it’s a short time, very short time, guys, the bridge has shortened, you understand? That’s the impression it gives. And I thought it was very nice because I was not used to walk towards the subway station. . I'm very happy, my physical strength has improved. From August until now I think I’ve improved almost 100 %.* | | | | | | | |
| 1D: I'm learning how to eat properly and make choices. | | | | | *That “doctor diet”- that you can-can’t eat -, when you leave the doctor already with an ideal weight, it’s done, everything that you couldn’t you eat. But now it’s not like this, you can eat a little, if I feel like eating some candy I eat... I'm learning how to eat properly. Like, many things: cookies, French-fries, those snacks, eliminate that. I’m learning how to choose what I eat, what I bring to my house, and that's the best thing. Since I’ve started the intervention I just ate and felt stomach pain, because of overeating, only twice. So the positive aspects are the nutritional and physical education; and the health, I improved a lot.* | | | | | | | |
| 1E: Before the intervention I used to stay alone in my house, now I retook many activities and I feel much better. | | | | | *This program was really good for me, because I used to stay home alone, so now I have to leave on Monday, Wednesday and Friday to come here. Well, I also studied here, but I kind have a relationship with the University... Amazingly, after the program everything has improved. In this meantime I recommenced talking to my supervisor, right, I’m finishing my bachelor’s degree, I resumed my academic life... Because I used to always speak with my supervisor, but I also disappeared, I used to say “I’ll be there in a while”. And now it’s different, I really go and I don’t feel guilty. I've noticed, since the beginning of the intervention, as I’m eating better, my skin, that had many spots when I got here, had already improved. I love staring at myself in the mirror. Now I don’ have as much pimples, because of the eating. I think that happiness is not in the weight loss, it is about feeling good. So if my “feel good” today is coming to college, doing my things, it may be part of my happiness.* | | | | | | | |
| 1F: I understood that the intervention is the kick off for changes. | | | | | *You have to understand that this intervention is for you kick off your healthy lifestyle, including a routine of activity and eating... I mean, we'll have to cut the umbilical cord, right?* | | | | | | | |
|  |  |  |  |  |  |  |  |  |  |  |  |  |
| **Table 2. Central ideas and collective subject discourse constructed from the discourses expressed in the third focus group (post 12 months), conducted with women who participated of a non-prescriptive multidisciplinary intervention based on the Health at Every Size® philosophy.** | | | | | | | | | | | | |
| **2) The intervention is coming to the end. What do you take from it?** | | | | | | | | | | | | |
| **CENTRAL IDEA** | | | | | **COLLECTIVE SUBJECT DISCOURSE** | | | | | | | |
| 2A: I take that every change is a process, is a diary learning. | | | | | *I think this one year intervention was for me to realize my limitations. It was a process. And the process is slow. It is a learning that we need to put into practice from day-to-day. I need to change my routine, to establish good routines, like exercising and nutritional education. And I'm just at the very beginning, I'm starting to crawl. Any distancing you let it go and then for you to start over you must have a very good judgment. It isn't over, it's something that you've learned today to put into practice for the rest of your life, it was very important.* | | | | | | | |
| 2B: I take a much better understanding of myself and of what I eat. | | | | | *I didn’t realize that sometimes food was a compensation for certain frustrations or certain situations that were not resolved. So, hey, if I’m excited I don’t need to eat, I need to spend energy, if I’m anxious I need to solve the problem, and not eat. So I have a better understanding of myself , of what I'm eating, why I'm eating, for what purpose I'm eating, the quality of what I'm eating, how I eat, how I ate. I started to analyze the food labels better, to exclude some things that still existed that didn’t make any difference regarding the flavor, but made ​​a big difference in the quality of what I was eating. And indeed, if we ate 20 years improperly and compensating situations with food, you will not solve it all in a year, but to be able to realize and say “I ate more than I should and wasn’t because I was hungry, it was because of other situation and I need now to notice myself not to do it again” I think it was a positive move.* | | | | | | | |
| 2C: I take a different expectation regarding my body. | | | | | *What do I take? I guess I won't be what I expected to be a year ago, like a very sexy woman, you know. I thought I would lose weight and I would become a blonde bombshell. And I found it very interesting because I had this imaginary and I think that was nice, this isn't the right way.* | | | | | | | |
| 2D: I take that it is possible to make changes. | | | | | *I learned that we can lose weight. I achieved a weight that I said “I won’t be able to come back anymore” and I did. I learned that it’s possible to remove at least half of what I ate before and I thought I wouldn’t. All right, sometimes you extrapolate, when it’s something that I do like a lot I end up eating, but then I know I won’t lack care you know, that I'm already pleased. And I left my job feeling very stressed. When I started the exercises, wow, it seemed that I felt a huge relieve. I learned that it is indeed possible to change our lives slightly. I made many changes that benefited my entire family. I should have taken a picture of my teenage son; he lost more weight than me. He criticized the nutritionist “I can’t believe that a person studies, dedicates herself to take care of someone’s eating”, but then with the changes I made in my habits, at the supermarket, he learned too, but my fitness is better than his.* | | | | | | | |
| 2E: I take learning and reminders about eating and physical activity. | | | | | *What was interesting that my nutritionist said was: there is nothing that fatten you up and nothing that slim you down. Because you’ll never stop eating a candy, you’ll never stop eating something that is pleasant to you. So well, it’s like this, you can restrain yourself today, but tomorrow is another story. Not to mention that you look at yourself, I think that it is your moment in the nutritional session. And I think there are several reminders, for example, I have the habit to eat breakfast standing up, then suddenly you remember the detail of sitting down. I started eating at every three hours, because I stay a long time without eating and when I eat I eat a lot. And many details we keep remembering about eating. That's nice because it reinforces, because if you drop it, you go back to square one, so I guess that these reminders must remain. And also what I'm taking from the intervention is: if I do some physical activity somewhere else, I'll know how to do the exercises, the preventions that I’ll have to take. So I think it was very important. Because it’s easy for you have weights at home but not know how to use them, and then you end up damaging yourself by other means.* | | | | | | | |
| **3) Today what do you consider as “success” in the treatment and what does it means for you?** | | | | | | | | | | | | |
| **CENTRAL IDEA** | | | | | **COLLECTIVE SUBJECT DISCOURSE** | | | | | | | |
| 3A: Success is to reflect about issues and realize our limitations | | | | | *This intervention, for sure, is related to some frustrations of our lives. It is an intervention that is really a psychological and physical treatment. You see people that assemble to you in some way. There were some participants I thought that had nothing to do with me, but today , for example, it has everything to do when she says that she’s not feeling that her body is okay because I feel that, even though my body is different from hers and maybe if I had her body it would be good for me, but the thought is very similar. So I see that being in this intervention has made us realize and reflect more about several things. With all of them, with the nutritionists, the physical educator professionals, the anthropologist, the philosopher, pulling something off from us we don’t stop to think about. And that's what ​​the intervention made: making you think where your issues are, your pendencies, what is leading you to eat. So to me it’s already a success, take from the program the perception you can realize, you can know you can do more. And also realize our limitations.* | | | | | | | |
| 3B: Success is being able to give continuity. | | | | | *It's being able to give continuity. Wake up in the morning and say: next week I don't have the nutritionist to orientate me, but I know that in the breakfast is like this, that if I eat better at every three hours I realized that's better for me. So that to me is already a success, try to carry on what I learned here.* | | | | | | | |
| **4) Making a self-assessment, how do you see yourselves today, at the end of the intervention? How do you imagine you will be a year from now?** | | | | | | | | | | | | |
| **CENTRAL IDEA** | | | | | **COLLECTIVE SUBJECT DISCOURSE** | | | | | | | |
| CI 4A: I see myself giving continuity to the physical activities. | | | | | *I think I'll turn myself to this matter of the physical activity more. I have never done it before and I discovered it’s not the worst thing in the world. It may not be the best, but I need it. So I see myself putting into practice what I have learned, that I think that really helped my family and I. The vigor is completely different, the rhythm, you ratiocinate better, so I see myself giving continuity: going out more, enjoying more of what the city has to offer. It has a library, a museum, you can walk to it and walk back, it has plenty of parks. It’s about maintaining the exercise, which is a routine we've been inserting, and maintaining the physical strength. Consequently we’ll lose weight, but it’s not the goal”* | | | | | | | |
| CI 4B: I see myself thinner and continuing what I’ve learned of nutrition. | | | | | *I see myself thinner in a year from. Because from now on, being able to lose weight and feeling well depend on me. Before I imagined that in order to lose weight I had to stop eating or eat a salad or only a meal per day. Today I know that I can eat a certain amount and it’s up to me knowing when to stop, because things will be available and we have to know what to choose, when, how much. This matter of the perception for me was essential, to realize how I ate, how I eat today”. So it’s to give continuity to that.* | | | | | | | |
| CI 4C: I see myself more empowered. | | | | | *I see myself more empowered to face some challenges. I want to go back to school, because until a year ago I thought it was impossible, but not now, I feel myself empowered for these things. I want to continue. I picture myself much better. Feeling good, you know? I want to feel good, with quality. I treat depression for about a million years, and I think I improved a lot this feeling of depression and it has been very well for myself. This intervention was the beginning for me to say: life goes on and things happen. So what I take is that every moment of your life you will face some things; if you want to lose weight you have to have a balance, a pattern, you won’t be able to eat everything.* | | | | | | | |
