# Supplementary material for: The Effects of a “Health at Every Size®”-Based Approach in Obese Women: A Pilot-Trial of the “Health and Wellness in Obesity” Study
Source: Front Nutr. 2015 Oct 27;2:34. doi: 10.3389/fnut.2015.00034 (PMC4621435; doi:10.3389/fnut.2015.00034)
Supplement: Supplementary file 2 [file Data_Sheet_1.DOCX]

***Supplementary Material***

**The effects of a ‘Health at Every Size®’-based approach in obese women: a pilot-trial of the ‘Health and Wellness in Obesity’ study**

Mariana Dimitrov Ulian^1^*, Fabiana B. Benatti^2^, Patricia Lopes de Campos-Ferraz^3^, Odilon J. Roble^4^, Ramiro Fernandez Unsain^5^, Priscila de Morais Sato^6^, Bruna Cristina Brito^1^, Karina Akemi Murakawa^2^, Bruno T. Modesto^2^, Luiz Aburad ^1^, Rômulo Bertuzzi^2^, Antonio H. Lancha Junior^2^, Bruno Gualano^2^, Fernanda B. Scagliusi^1^.

^1^ Department of Nutrition, Faculty of Public Health, University of Sao Paulo, Sao Paulo, SP, Brazil.

^2^ School of Physical Education and Sport, University of Sao Paulo, Sao Paulo, SP, Brazil.

^3^ Faculty of Applied Sciences, State University of Campinas, Limeira, SP, Brazil.

^4^ Faculty of Physical Education, State University of Campinas, Campinas, SP, Brazil.

^5^ Faculty of Philosophy and Letters, National University of Buenos Aires, Buenos Aires, Argentina.

^6^ Institute of Health and Society, Federal University of Sao Paulo, Santos, SP, Brazil.

***Address correspondence to**: Mariana Dimitrov Ulian, Department of Nutrition, University of Sao Paulo, Faculty of Public Health, Av. Dr. Arnaldo, 715, Sao Paulo, SP, Zip Code: 01246-904, Brazil. Phone: 55 11 3061-7755. Email: m.dimitrov@usp.br.

# Supplementary Data

**Professionals’ training and contributions**

Before commencing the intervention, the physical education, philosophy, and nutrition professionals had 60 hours of interdisciplinary meetings about non-prescriptive interventions. These trainings were facilitated by one of the authors of this article (FBS) and the topics of discussion were defined in advance by the coordinators of the intervention. In these meetings, all professionals exposed their frames of references, and scientific references (related to non-prescriptive interventions and interdisciplinary experiences, such as Carvalho and Martins (1), Bacon (2), Brasil (3), Castiel and Álvarez-Dardet (4), Sallis (5) were used to prompt reflections and discussions. Additionally, the nutritional therapists underwent 30 hours of training in nutritional counseling techniques and principles. These trainings were also facilitated by the author FBS, who has clinical and teaching experience with nutritional counseling. The training curriculum was composed of expository lessons and that focused on theories and models that aimed to offer systematic explanations for nutrition-related behavior changes, and presented the theoretical framework and strategies used in the nutrition counseling (e.g., the establishment of rapport and of a collaborative relationship between the nutritional therapist and the participant; dealing with eating attitudes, that is, beliefs, thoughts, feelings and behaviors concerning food; the establishment of goals, the creation of individualized action plans, the goal setting) with practical case-studies exercises.

Each of the involved professionals were essential to the viability of the intervention due to their distinct characteristics and roles: the physical activity professionals aimed to help participants to find joy in moving their bodies and were trained to have a nonjudgmental attitude regarding the participants’ performance; the nutritional therapists aimed to help the participants to establish a healthier and more meaningful relationship towards eating (if and when necessary) and; the philosopher aimed to establish an environment where participants could feel welcome and comfortable to speak what they felt like.

**References**

1. Carvalho MC, Martins A. A obesidade como objeto complexo: uma abordagem filosófico-conceitual. *Ciênc saúde coletiva* (2004) **9**(4):1003–1012. http://dx.doi.org/10.1590/S1413-81232004000400021.
2. Bacon L. *Health at Every Size: the surprising truth about your weight*. Dallas,Texas, Benbella Books, 2010.
3. Brasil. *Clínica ampliada e compartilhada*. Brasília, DF: Ministério da Saúde, 2009.
4. Castiel LD, Álvarez-Dardet C. *A saúde persecutória: os limites da responsabilidade*, Rio de Janeiro, Fiocruz, 2007.
5. Sallis RE. Exercise is medicine and physicians need to prescribe it! *Br J Sports Med* (2009) **43**(1):3–4.
